# A novel phosphorylation site in SARS-CoV-2 nucleocapsid regulates its RNA-binding capacity and phase separation in host cells

**DOI:** 10.1093/jmcb/mjac003

**Published:** 2022-01-18

**Authors:** Junyu Wu, Yongheng Zhong, Xue Liu, Xiaoyu Lu, Weijie Zeng, Chunyan Wu, Fan Xing, Liu Cao, Fuxiang Zheng, Panpan Hou, Hong Peng, Chunmei Li, Deyin Guo

**Affiliations:** Centre for Infection and Immunity Study (CIIS), School of Medicine, Shenzhen Campus of Sun Yat-sen University, Shenzhen 518197, China; Centre for Infection and Immunity Study (CIIS), School of Medicine, Shenzhen Campus of Sun Yat-sen University, Shenzhen 518197, China; Centre for Infection and Immunity Study (CIIS), School of Medicine, Shenzhen Campus of Sun Yat-sen University, Shenzhen 518197, China; Centre for Infection and Immunity Study (CIIS), School of Medicine, Shenzhen Campus of Sun Yat-sen University, Shenzhen 518197, China; Centre for Infection and Immunity Study (CIIS), School of Medicine, Shenzhen Campus of Sun Yat-sen University, Shenzhen 518197, China; Centre for Infection and Immunity Study (CIIS), School of Medicine, Shenzhen Campus of Sun Yat-sen University, Shenzhen 518197, China; Centre for Infection and Immunity Study (CIIS), School of Medicine, Shenzhen Campus of Sun Yat-sen University, Shenzhen 518197, China; Centre for Infection and Immunity Study (CIIS), School of Medicine, Shenzhen Campus of Sun Yat-sen University, Shenzhen 518197, China; Centre for Infection and Immunity Study (CIIS), School of Medicine, Shenzhen Campus of Sun Yat-sen University, Shenzhen 518197, China; Centre for Infection and Immunity Study (CIIS), School of Medicine, Shenzhen Campus of Sun Yat-sen University, Shenzhen 518197, China; Centre for Infection and Immunity Study (CIIS), School of Medicine, Shenzhen Campus of Sun Yat-sen University, Shenzhen 518197, China; Centre for Infection and Immunity Study (CIIS), School of Medicine, Shenzhen Campus of Sun Yat-sen University, Shenzhen 518197, China; Centre for Infection and Immunity Study (CIIS), School of Medicine, Shenzhen Campus of Sun Yat-sen University, Shenzhen 518197, China

**Dear Editor**,

The severe acute respiratory syndrome coronavirus 2 (SARS-CoV-2) is a novel emerging coronavirus that has spread worldwide since breaking out in late 2019 and has led to hundreds of millions of infections and millions of human deaths (Zhou et al., 2020). The genome of SARS-CoV-2 encodes 29 viral proteins, including four structural proteins: spike (S), envelope (E), membrane (M), and nucleocapsid (N) (Kim et al., 2020). N protein is essential for viral genomic RNA replication and packaging and it also plays an important role in the virus‒host interactions (Lang et al., 2021). The amino acid sequences of N protein are highly conserved among coronaviruses (Supplementary Figure S1). Accumulating evidence indicates that N protein is a phosphoprotein and its phosphorylation state is important for its proper function (Peng et al., 2008; Wu et al., 2014).

Here, we try to map the phosphorylation sites in N protein of SARS-CoV-2 and investigate their functions. Flag-tagged N protein was expressed in HEK293T cells and the phosphorylation level of N protein was detected by immunoprecipitation followed by western blotting assay. As expected, the phosphorylation can be easily detected by anti-phospho-serine (S)/threonine (T) antibody (Figure 1A), implying that the N protein is highly phosphorylated in cells. Then, a large amount of N protein was immunoprecipitated and analyzed by mass spectrometry technology (Figure 1B). The results showed that 71% of the N protein sequence was covered and 15 phosphorylated sites were detected (Figure 1C; Supplementary Figure S2 and Table S1). Some peptides were phosphorylated on a single site, while the others appeared to have multiple phosphorylated sites. Intriguingly, the ‘GFYAEGSRGGSQASSR’ peptide showed different phosphorylation profiles on S176/S180/S183/S184 (Supplementary Figure S2 and Table S1), implying that it is highly phosphorylated in cells. After mapping along the protein sequence, we found that most of the phosphorylation sites are located in the arginine/serine (SR)-rich region (Figure 1D), which has been reported to be the highly phosphorylated region of SARS-CoV (Peng et al., 2008). Apart from the SR-rich region, there are also a few phosphorylation sites residing in the N-terminal domain (NTD) and the C-terminal domain (CTD) (Figure 1D). The NTD of N protein is also named the RNA-binding domain, which is responsible for viral RNA binding, while the CTD is also called the homodimerization domain, which is responsible for N protein dimerization. The CTD of N protein has also been documented to bind with viral RNA (Ye et al., 2020). Thus, the phosphorylation on the NTD or CTD may regulate their proper function.

**Figure 1:**
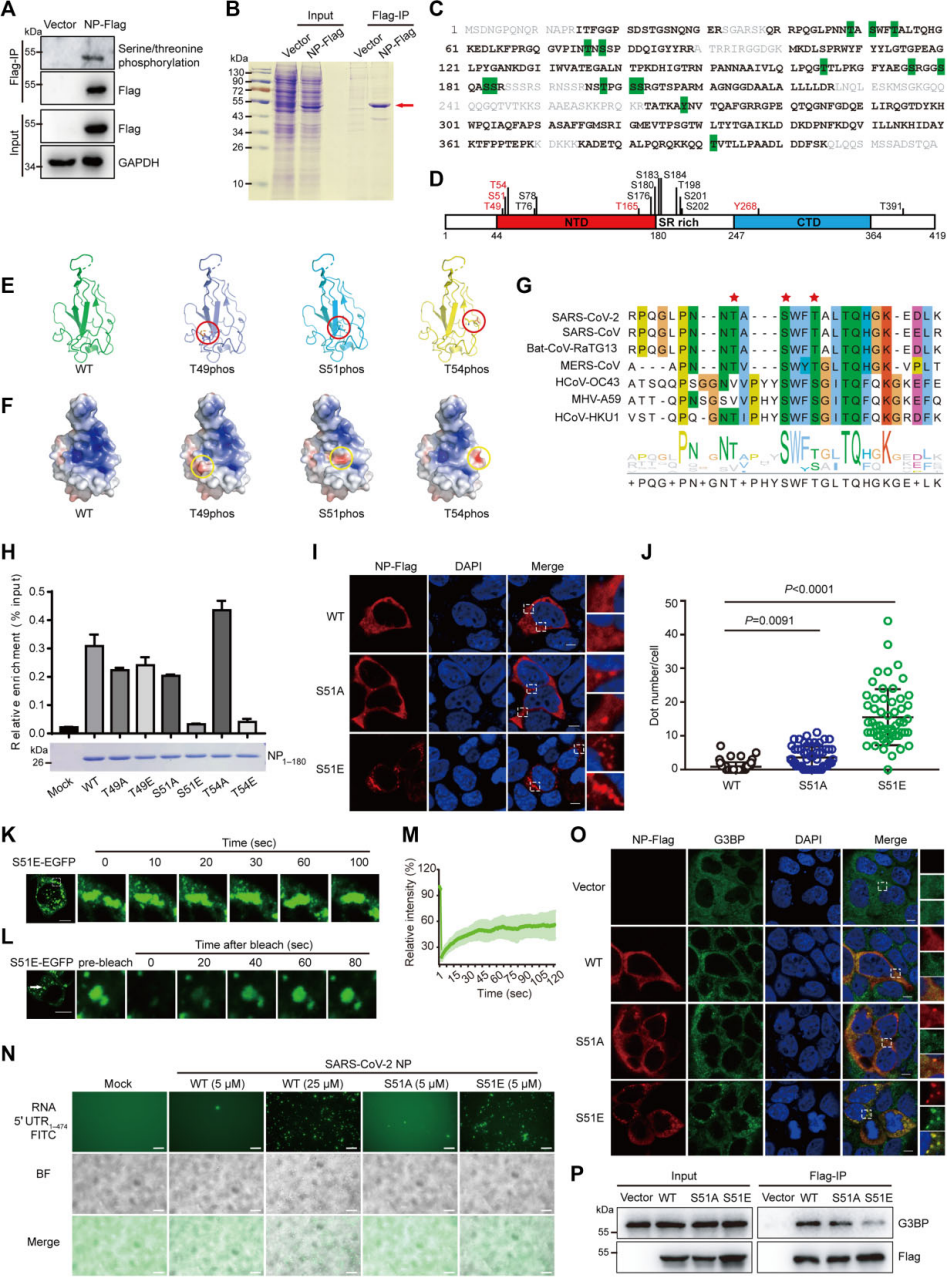
A novel phosphorylation site in the RNA-binding pocket of SARS-CoV-2 N protein regulates its RNA-binding capacity and phase separation. (**A**) N protein is phosphorylated in cells. C-terminal Flag-tagged N protein of SARS-CoV-2 (N-Flag) was expressed in HEK293T cells for 48 h and then immunoprecipitated by anti-Flag beads. The phosphorylation states of N protein were detected by using a pan-phospho-serine/threonine antibody. (**B**) Sodium dodecyl sulfate‒polyacrylamide gel electrophoresis and Coomassie brilliant blue staining of N protein purified from HEK293T cells. (**C** and **D**) Phosphorylation sites detected in N protein. Purified N protein was digested by trypsin and further analyzed by liquid chromatography tandem mass spectrometry. (**C**) The detected tryptic peptides are marked in black and bold, while the uncovered residues are marked in gray along the protein sequence of N protein. Observed phosphorylation sites are shaded in green. (**D**) The positions of the observed phosphorylation sites are represented in the schematic diagram of N protein. The previously unreported phosphorylation sites are marked in red. (**E** and **F**) Phosphorylated residues near the RNA-binding pocket in the NTD of N protein. (**E**) Phosphorylated residues (red circle) in the tertiary structure of NTD of N protein (PDB: 6M3M). (**F**) The electrostatic potential surface changes of the nonphosphorylated and phosphorylated NTD of N protein. Electrostatic potentials were calculated with the APBS plug-in of PyMol and the electrostatic surface is colored continuously from red to blue (−5.0 kT/e to +5.0 kT/e). Positions of the related phosphorylation sites are indicated by the yellow circles. (**G**) Multiple sequence alignment of the RNA-binding pocket in the NTD of N protein across different coronaviruses. Asterisks indicate the identified phosphorylation sites. (**H**) The phosphorylation mimic mutations, S51E and T54E, block the RNA-binding capacity of the NTD in N protein. The recombinant NTD domain of N protein (residues from 1 to 180) with the indicated mutations were incubated with the *in vitro* transcribed 5′ UTR of viral RNA for 30 min at 37°C. The viral RNA bond with these proteins was precipitated by Ni-NTA μsphere agarose beads and further quantified by reverse transcription–quantitative PCR assay. The graph represents mean ± SD from three independent replicates measured in triplicate. (**I** and **J**) The S51E mutant of N protein forms puncta in cells. (**I**) Caco-2 cells were transfected with the indicated plasmids and then fixed and stained with anti-Flag antibody (red) and DAPI (blue) after 24 h. Scale bar, 10 μm. (**J**) Statistical analysis of the puncta numbers formed by N protein mutants. Two independent experiments were performed, and representative data are shown as mean ± SD (*n* = 63, 59, and 56, respectively). Statistics were calculated by the Mann‒Whitney *U* test. (**K‒M**) The LLPS property of S51E mutant puncta in cells. S51E-EGFP plasmid was expressed in 293T cells for 24 h. (**K**) The fusion of S51E-EGFP puncta was observed in the cytoplasm of cells. (**L**) The fluorescence of S51E-EGFP puncta recovers partially after photobleaching. (**M**) Following 5 sec of photobleaching, images were taken every 1 sec and the relative fluorescence intensity to pre-bleaching intensity was calculated (mean ± SD, *n* = 7). (**N**) S51E mutation promotes phase separation of N protein *in vitro*. The indicated concentrations of N proteins (WT, S51A, or S51E) were mixed with FITC-labeled 5′ UTR of viral RNA at 37°C. Fluorescence and bright-field (BF) images were taken after 20 min of incubation. Scale bar, 10 μm. (**O**) The S51E mutant of N protein induces the formation of SGs. Caco-2 cells were transfected with the WT, S51A, or S51E mutant N protein, respectively. Cells were then fixed and coimmunostained with anti-Flag (red) and anti-G3BP (green) antibodies. Nuclei were stained by DAPI (blue). Scale bar, 10 μm. (**P**) S51E mutation weakens the interaction between N protein and G3BP. HEK293T cells transfected with the indicated N protein mutants were subjected to immunoprecipitation with anti-Flag antibody. The immunoprecipitated G3BP was assessed by western blotting.

We integrated our results with the reported phosphorylation sites of N protein in different experimental settings (Supplementary Figure S3A). In total, 38 amino acid residues in N protein were found to be phosphorylated and, consistently, they were enriched in the SR-rich region (Supplementary Figure S3B). Then, a total of 57613 high-quality SARS-CoV-2 genome sequences before 20 August 2020 were downloaded from the Global Initiative on Sharing All Influenza Data (GISIAD) and the mutation frequencies of these phosphorylation sites in N protein were analyzed. As shown in Supplementary Tables S2 and S3, the phosphorylation sites in the SR-rich region mutated more frequently, while the sites in the NTD and CTD regions were very stable with few mutation records.

Comparing with previous reports, our study detected five novel phosphorylation sites, T49, S51, T54, T165, and tyrosine (Y)268, respectively (Figure 1D; Supplementary Figure S3A). Intriguingly, T49, S51, and T54 are situated in the RNA-binding pocket of the NTD domain (Figure 1E). They are highly stable in the SARS-CoV-2 genome and are conserved in the beta-coronavirus (Figure 1G; Supplementary Table S2). As the RNA binding of NTD relies on its positive-charged surface to associate with the negative-charged phosphate skeletons of RNA (Kang et al., 2020), the phosphorylation on T49, S51, or T54 may change the charge surface of NTD and consequently affect its RNA-binding capacity. Then, we calculated the change of the charge surface with PyMOL software. As expected, phosphorylation of these three sites apparently changed the surface charge state of NTD (Figure 1F). To further gain insight into the impact of NTD phosphorylation on its RNA binding, the S/T residues were mutated to glutamic acid (E) or alanine (A) to mimic the phosphorylated or unphosphorylated states, respectively. As both the NTD and CTD have been reported to bind with viral RNA, to get rid of effects of CTD, the His-tagged recombinant wild-type (WT) or mutant NTD domains of N protein (1‒180 amino acids) were expressed, purified, and then incubated with the *in vitro*-transcribed 5′ untranslated region (UTR) of viral RNA. The *in vitro* RNA-binding assay showed that the phosphorylation mimic mutations, S51E and T54E, dramatically blocked the RNA-binding capacity of the NTD domain (Figure 1H).

We further evaluated the influence of phosphorylation in the NTD domain. The full-length WT or mutant N proteins were transfected into Caco-2 cells and the distribution was visualized by immunostaining. Similarly to the WT N protein, T49A, T49E, T54A, and T54E mutant proteins diffused in cytoplasm (Figure 1I; Supplementary Figure S4). However, S51E mutant protein formed apparent protein aggregates and S51A mainly diffused with a few aggregates in cytoplasm (Figure 1I and J). Similarly, discrete green puncta were observed in the cytoplasm of HEK293T cells expressing S51E-enhanced green fluorescent protein (EGFP) fusion protein (Supplementary Figure S5). Recent evidence indicates that N protein of SARS-CoV-2 undergoes liquid–liquid phase separation (LLPS) to form condensates with viral RNA (Carlson et al., 2020; Chen et al., 2020). Carlson et al. (2020) demonstrated that the phosphorylation in the SR-rich region promotes the LLPS of N protein by using a phosphomimetic mutant replacing the 10 S/T residues in the SR region with aspartate. In view of the apparent protein aggregates formed by the S51E mutant in cells, we wondered whether the single phosphorylation at the S51 site also promotes the LLPS of N protein. The fusion of S51E-EGFP puncta was observed in the cytoplasm of cells (Figure 1K) and the fluorescence of S51E-EGFP puncta recovered partially after photobleaching (Figure 1L and M), indicating the dynamic recruitment of fluorescence protein. These results suggest the liquid-like property of S51E-EGFP puncta in cells. To further evaluate this property, the full-length WT, S51A, and S51E mutant N proteins were recombinantly expressed and purified. Consistent with the previous reports, the WT N protein (≥25 μM) underwent phase separation with viral RNA (Supplementary Figure S6). Intriguingly, S51E mutant protein formed liquid-like droplets at lower concentration (5 μM) (Figure 1N; Supplementary Figure S7A) and blocked the migration of viral RNA in the agarose gel (Supplementary Figure S7B). In line with the results in cells, the fluorescence of these droplets recovered partially in fluorescence recovery after photobleaching assays (Supplementary Figure S8). Taken together, these results demonstrated that phosphorylation at the S51 site in the NTD of N protein promotes its LLPS with viral RNA.

Stress granules (SGs) are nonmembranous mRNA‒protein aggregates, which can be induced by viral infection and function to restrict viral proliferation (McCormick and Khaperskyy, 2017). Previous proteomic study has identified that SARS-CoV-2 N protein associates with G3BP1/2, the core components of SG (Gordon et al., 2020). Then, we evaluated the relationship of N protein mutants with G3BP proteins. As shown in Figure 1O and Supplementary Figure S9, the WT, T49A, T49E, S51A, T54A, and T54E mutant proteins did not trigger the formation of SGs, but they were colocalized with G3BP signals in a diffused state, which is consistent with the previous reports that N protein of SARS-CoV-2 sequesters G3BP and disrupts SG formation (Zheng et al., 2021). However, G3BP proteins formed apparent protein aggregates with S51E mutant protein (Figure 1O), indicating that phosphorylation in S51 released the inhibition of SG formation. We further investigated the interaction of N protein with G3BP by immunoprecipitation. As shown in Figure 1P, N protein indeed interacted with G3BP proteins, while such interaction was weakened in the S51E mutant. Altogether, our results suggested that phosphorylation at the S51 site attenuates the interaction with G3BP proteins and releases its inhibition in the SG formation.

In summary, our study demonstrate that N protein of SARS-CoV-2 is highly phosphorylated in host cells and finds several novel phosphorylation sites, among which the phosphorylation on S51 resides directly at the center of the RNA-binding pocket of the N protein NTD. The phosphorylation mimicry study suggests that S51 phosphorylation may inhibit the RNA-binding capacity of the NTD domain and further promote the phase separation of N protein and release its inhibition in the SG formation. Further efforts are needed to find out the kinase/phosphatase of this site and illustrate its detailed regulatory mechanism in the future. Also, it will be interesting to explore the influence of S51 phosphorylation on viral RNA replication or virion packaging with a live virus system in the high-level biosafety laboratory.

*[This study was supported by the National Natural Science Foundation of China (32041002 to D.G. and 31800151 to J.W.), Guangdong Province ‘Pearl River Talent Plan’ Innovation and Entrepreneurship Team Project (2019ZT08Y464 to C.L.), and Shenzhen Science and Technology Program (JSGG20200225150431472 and KQTD20180411143323605 to D.G.; GXWD20201231165807008 and 20200825183117001 to J.W.). D.G. is also supported by Guangdong Zhujiang Talents Program and National Ten-thousand Talents Program. D.G. and J.W. designed the project; J.W. and Y.Z. performed most of the experiments; X. Liu, X. Lu, W.Z., C.W., F.X., L.C., F.Z., P.H., H.P., and C.L. provided technical assistance; D.G. and J.W. analyzed data and wrote the manuscript.]*

## Supplementary Material

mjac003_Supplemental_File
