# Supplementary material for: A novel phosphorylation site in SARS-CoV-2 nucleocapsid regulates its RNA-binding capacity and phase separation in host cells
Source: J Mol Cell Biol. 2022 Jan 18;14(2):mjac003. doi: 10.1093/jmcb/mjac003 (PMC9122633; doi:10.1093/jmcb/mjac003)
Supplement: mjac003_Supplemental_File [file mjac003_supplemental_file.pdf]

Supplementary material

A novel phosphorylation site in SARS-CoV-2 nucleocapsid regulates its RNA-binding capacity and phase separation in host cells

Junyu Wu[†,*], Yongheng Zhong[†], Xue Liu, Xiaoyu Lu, Weijie Zeng, Chunyan Wu, Fan Xing, Liu Cao, Fuxiang Zheng, Panpan Hou, Hong Peng, Chunmei Li, and Deyin Guo*

Centre for Infection and Immunity Study (CIIS), School of Medicine, Shenzhen Campus of Sun Yat-sen University, Shenzhen 518197, China

[†]These authors contributed equally to this work.

*Correspondence to: Deyin Guo, Tel: +86 020-87335462; Fax: +86 020-87335462; E-mail: guodeyin@mail.sysu.edu.cn; Junyu Wu, E-mail: wujy68@mail.sysu.edu.cn

Materials and Methods

Plasmid construction

The plasmid encoding full-length SARS-CoV-2 N protein was a kind gift from Prof. Pei-Hui Wang (Shandong University, China). We subcloned the N gene into pHAGE-CMV-Flag and pEGFP-N1 expression vector. The S49A, S49E, S51A, S51E, S54A, S54E mutants were generated by using site-directed mutagenesis PCR with PrimeSTAR GXL DNA Polymerase (TAKARA, R050A). The full-length and NTD domain (residues 1-180) of N protein, including the wild type and mutants, were subsequently cloned into pET-30a (+) vector for recombinant expression. All the plasmids were validated by sequencing. The primers are listed in the Supplementary information, Table S4.

Cell culture

Caco-2 cells and HEK293T cells were maintained in Dulbecco's Modified Eagle Medium (DMEM, Corning, 10-013-CVRC) with 10% fetal bovine serum (FBS, Gibico, 10270-106) and 1% Penicillin-Streptomycin Solution (P/S, Gibico, 15140-122) in a 5% CO2 incubator at 37 ℃.

Plasmid transfection

HEK293T cells were seeded in 10 cm dishes 24h prior to transfection. The plasmids were transfected into cells at 80% confluence with Lipofectamine™ 2000 Transfection Reagent (Invitrogen, 11668019) following manufacturer’s instructions. Caco-2 cells were transfected in suspension with plasmids using Lipofectamine™ 3000 Transfection Reagent (Invitrogen, L3000015) according to the manufacturer’s protocol.

Immunoprecipitation

HEK293T cells were harvested at 48h post transfection. After rinsed with ice-cold PBS, cells were lysed in IP buffer (50 mM Tris-HCl, pH7.4, 150 mM NaCl, 10% Glycerol, 1% NP40, 1 mM DTT) supplemented with cOmplete Protease Inhibitor Cocktail Tablets (Roche,11697498001) and PhosSTOP phosphatase inhibitor cocktail Tablets (Roche, 4906845001). The cell lysates were centrifuged at 12000g for 15 min at 4 ℃. The supernatants were then incubated with anti-Flag magnetic beads (Bimake, B26102) at 4 ℃ for 2h. After three times of thorough washing with IP buffer, the flag-tagged N protein was eluted with Poly FLAG peptide (Bimake, B23111). The immunoprecipitated samples were then subjected to Western blotting or mass spectrometry analysis.

Western blotting analysis

Western blotting was performed as described in our previous report(He et al., 2016). Briefly, the cell lysates or immunoprecipitates were boiled with SDS loading buffer for 10 min and then were separated on 10% SDS-PAGE gel. Proteins were transferred onto methanol-activated PVDF membrane (Millipore, IPVH00010). After blocked with 5% skim milk, the membrane was probed with antibodies as indicated and then were visualized with Clarity^{TM} Western ECL substrate (Bio-Rad, 1705061) by using the Tanon-5200Multi Gel Imaging System (Tanon Science & Technology, China). Antibodies used in this study: Anti-FLAG M2 Antibody (Sigma-Aldrich, F1804), anti-G3BP (Abcam, ab56574), anti-GAPDH (Proteintech, 10494-1-AP), anti-Phosphoserine (abcam, ab9332), Horseradish peroxidase (HRP) conjugated anti-mouse&rabbit (Abmart, M21003).

Mass spectrometry analysis

The immunoprecipitates were separated on 10% SDS-PAGE gel and were stained with 0.1% Coomassie Brilliant Blue R250 (Sigma, 1125530025). The target band of approximately 50 kD was excised and sent to the Guangzhou FitGene Biotechnology Co. Ltd for trypsin digestion and further HPLC-MS/MS analysis.

Immunofluorescent staining and microscopy

Caco-2 cells were transfected in suspension and seeded onto the 18 mm Microscope Cover Glass (Fisherbrand, 12542A). Twenty-four hours later, cells were fixed with 4% paraformaldehyde for 15 min at room temperature. After permeabilized by 0.2% Triton X-100 and blocked with 3% BSA (Abcone, B24726), the coverslips were incubated overnight with the indicated primary antibodies at 4 ℃. Cells were washed with PBST (PBS plus 0.1% Tween-20) for three times and subsequently incubated with Donkey anti-Mouse IgG (H+L) Highly Cross-Adsorbed Secondary Antibody, Alexa Fluor 488 (ThermoFisher, A21202) and Donkey anti-Rabbit IgG (H+L) Highly Cross-Adsorbed Secondary Antibody, Alexa Fluor 555 (ThermoFisher, A31572) at room temperature for 1 h and with DAPI for 5 min. The mounted coverslips were then imaged using Confocal Microscope (Zeiss LSM 880) equipped with a 63× oil immersion objective.

Recombinant protein expression and purification

The pET-30a (+) expression plasmids encoding the wild type and mutants of full-length or the NTD OF N protein were transformed into *Escherichia coli* BL21 (DE3). Bacterial cells were grown in fresh LB medium containing kanamycin (50 μg/ml) to about 0.8 OD600 at 37 °C and then were induced with 1 mM isopropyl-β-D-thiogalactopyranoside (IPTG, Solarbio, I1020-5) at 16 ℃ overnight. Cells were collected by centrifugation and were resuspended in lysis buffer (1× PBS, 20 mM imidazole, 6 M urea, 1× protease inhibitors). After sonication on ice, cell lysates were centrifuged at 12,000 g at 4 ℃ for 20 min and the supernatants were incubated with Ni-NTA μSphere agarose beads (Goodbio, DG-0104) for 2h in 4 ℃. The beads were washed with the lysis buffer supplemented with 50 mM imidazole and then the proteins were eluted with elution buffer (1× PBS, 500 mM imidazole, 6 M urea, 1× protease inhibitors). The purified proteins were refolded by dialysis in PBS overnight in 4 ℃ and further concentrated using Amicon Ultra 30K or 10K filter units (Millipore, UFC803096, UFC801096). After quantified by the BCA kit (Pierce, 23225), the proteins were aliquoted, flash-frozen and stored at -80 ℃.

***In vitro* RNA transcription**

The 5'UTR (1-474nt) of SARS-CoV-2 genomic RNA was *in vitro* transcribed for the RNA binding and phase separation experiments. DNA templates were amplified from the cDNA of SARS-CoV-2 infected Vero E6 cells. The *in vitro* transcription was carried out by using the *in vitro* MEGAscript™ T7 Transcription Kit (Invitrogen, AMB13345) with the addition of Fluorescein RNA Labeling Mix (Roche, 11685619910) for 3h at 37 ℃. The products were treated with DNase I digestion for 30 min and further purified according to the manufactures' instructions. The purity and size of RNA was verified by denaturing agarose gel and the RNA concentration was measured by using nanodrop.

***In Vitro* RNA pulldown assay**

The His-tagged recombinant wild-type or mutant NTD domains of N protein (1-180 aa) were expressed in *Escherichia coli* BL21 (DE3) and purified as described above. Two micrograms of the recombinant proteins were incubated with five micrograms of the *in vitro*-transcribed 5' UTR of viral RNA in binding buffer (25 mM Tris-HCl pH7.0, 150 mM KCl, 5 mM MgCl2, 0.5 mM dithiothreitol, 5 mM EDTA) with 40 U RNasin® Ribonuclease Inhibitor (Promega, N2511) for 30 min at 37 ℃. Then, Ni-NTA μSphere agarose beads were added into the RNA-protein mix and incubated for another 30 min at 4 ℃. After five times of thorough wash with the binding buffer, the viral RNA bond with these proteins on beads was purified with the TRIZol reagent according to the manufactures' instructions. The level of precipitated viral RNA was further quantified by RT-qPCR assay with the primers listed in the Supplementary information, Table S4.

Gel mobility shift assay

The agarose gel mobility shift assay was carried out as previously described(Ream et al., 2019). The *in vitro* transcribed RNA (about 4 μg) was incubated with increasing concentrations of wild type or mutants of full-length N proteins (0, 0.625, 1.25, 2.5, 5 μM) in binding buffer (25 mM Tris-HCl pH7.0, 150 mM KCl, 5

mM MgCl2, 0.5 mM dithiothreitol, 5 mM EDTA) with 40 U RNasin® Ribonuclease Inhibitor (Promega, N2511) for 30 min at 37 ℃. Samples were loaded onto the 1.5% agarose gel and run for 40 min at 130 V. The bound and free RNAs were detected at wavelength of 302 nm by using the Tanon-5200Multi Gel Imaging System (Tanon Science & Technology, China).

***In vitro* phase separation assays**

To validating the phase separation activity of N protein, an increasing concentrations of wild type N protein (0, 5, 10, 25, 50 μM) were mixed with 4μg FITC-labeled 5'UTR viral RNA in PBS (pH7.4) and incubated for 20 min at 37 ℃. Five microliters of each sample were loaded onto a 3.5 cm glass bottom dish. Imaging was performed with Axio Observer Z1 microscopy (Zeiss) equipped with a 63× oil immersion objective.

To investigate the influence of S51 phosphorylation on phase separation, 5 μM of wild type, S51E, S51A mutant N proteins were incubated with 4μg FITC-labeled viral RNA, separately. Imaging was performed as described above.

Fluorescence recovery after photobleaching (FRAP) in cells or *in vitro*

The FRAP experiments were performed on Nikon A1R confocal laser scanning microscope. For *in vitro* FRAP assay, The WT (25 μM) or S51E mutant (5 μM) N protein were incubated with FITC-labeled 5' UTR of viral RNA and incubated for 20 min at 37 ℃. Then, fifteen microliters of each sample were loaded onto a 3.5 cm glass bottom dish. Two images were acquired before photobleaching. The selected droplets were photobleached with 10% laser power for 5 s using 488 nm lasers and time-lapse images were taken every 10 s for over 3 minutes after bleaching. Cellular FRAP was performed with S51E-EGFP expressing HEK293T cells with the same photobleaching parameters at 37 °C in a live-cell-imaging chamber. Time-lapse images were taken every 1s for over 2 minutes after bleaching. Images were analyzed by ImageJ and the FRAP recovery curves were fitted using GraphPad Prism 7.

Molecular modelling of N protein phosphorylation sites

The X-ray crystallographic structure of N protein of SARS-CoV-2 (PDB ID code: 6M3M) was obtained from the Protein Data Bank. Molecular modeling of phosphorylation at T49, S51 or T54 were performed using PyMOL software (http://www.pymol.org). The electrostatic potential molecular surface of these phosphorylation sites was calculated and represented by using the Advanced Poisson-Boltzmann Solver (APBS) plugin in PyMOL.

RNA extraction, reverse transcription and quantitative real-time PCR (qRT-PCR)

Total cellular RNAs were extracted by using TRIzol reagent (ThermoFisher Scientific, 15596026) and were then reverse transcribed into cDNA with PrimeScript RT Reagent Kit with gDNA Eraser (Takara, RR047A) following the manufacturer's instructions. qRT-PCR was performed using PowerUp SYBR Green Master Mix (ThermoFisher Scientific, A25742) with the ABI QuantStudio5 (Applied Biosystems, USA). The relative expression level of target RNAs were normalized to GAPDH housekeeping gene. The primers were

listed in Supplementary information, Table S4.

Mutation frequency analysis

To analyze the mutation frequency of the phosphorylation sites in N-protein, a total of 57613 high-quality SARS-CoV-2 genome sequences were downloaded from the Global Initiative on Sharing All Influenza Data (GISIAD)(Shu and McCauley, 2017) as of 20 August 2020. Multiple sequences alignments were performed using the progressive method (FFT-NS-2) implemented in MAFFT (version 7.4)(Katoh et al., 2002). The mutation analysis was carried out by using the pipeline provided by CoVa (version 0.2) with the NC_045512.2 SARS-CoV-2 genome(Wu et al., 2020) as reference sequence.

Statistical analyses

Quantitative data were presented as means ± standard deviation (SD). Statistical analyses were performed with the GraphPad Prism 7.0.0 software (GraphPad Software, Inc.). Significance was assessed with the non-parametric Mann–Whitney U-test. If $p < 0.05$, the difference was considered as significant.

Data availability

All data supporting the findings of this study are included in the article and/or its supplementary information. The relevant materials are available from the corresponding author upon reasonable request.

Reference

Bouhaddou, M., Memon, D., Meyer, B., et al. (2020). The Global Phosphorylation Landscape of SARS-CoV-2 Infection. Cell*, 182*, 685-712 e619.

Davidson, A. D., Williamson, M. K., Lewis, S., et al. (2020). Characterisation of the transcriptome and proteome of SARS-CoV-2 reveals a cell passage induced in-frame deletion of the furin-like cleavage site from the spike glycoprotein. Genome Med*, 12*, 68.

He, Z., Wu, J., Su, X., et al. (2016). JMJD5 (Jumonji Domain-containing 5) Associates with Spindle Microtubules and Is Required for Proper Mitosis. J Biol Chem*, 291*, 4684-4697.

Jack, A., Ferro, L. S., Trnka, M. J., et al. (2020). SARS CoV-2 nucleocapsid protein forms condensates with viral genomic RNA. bioRxiv.

Katoh, K., Misawa, K., Kuma, K., and Miyata, T. (2002). MAFFT: a novel method for rapid multiple sequence alignment based on fast Fourier transform. Nucleic Acids Res*, 30*, 3059-3066.

Klann, K., Bojkova, D., Tascher, G., Ciesek, S., Munch, C., and Cinatl, J. (2020). Growth Factor Receptor Signaling Inhibition Prevents SARS-CoV-2 Replication. Mol Cell*, 80*, 164-174 e164.

Ream, J. A., Lewis, L. K., and Lewis, K. A. (2019). Horizontal Agarose Gel Mobility Shift Assay for Protein-RNA Complexes. Methods Mol Biol*, 1855*, 363-370.

Shu, Y., and McCauley, J. (2017). GISAID: Global initiative on sharing all influenza data - from vision to reality. Euro Surveill*, 22*.

Supekar, N. T., Shajahan, A., Gleinich, A. S., et al. (2021). Variable post-translational modifications of SARS-CoV-2 nucleocapsid protein. Glycobiology.

Wu, F., Zhao, S., Yu, B., et al. (2020). A new coronavirus associated with human respiratory disease in China. Nature*, 579*, 265-269.

Supplementary Figures

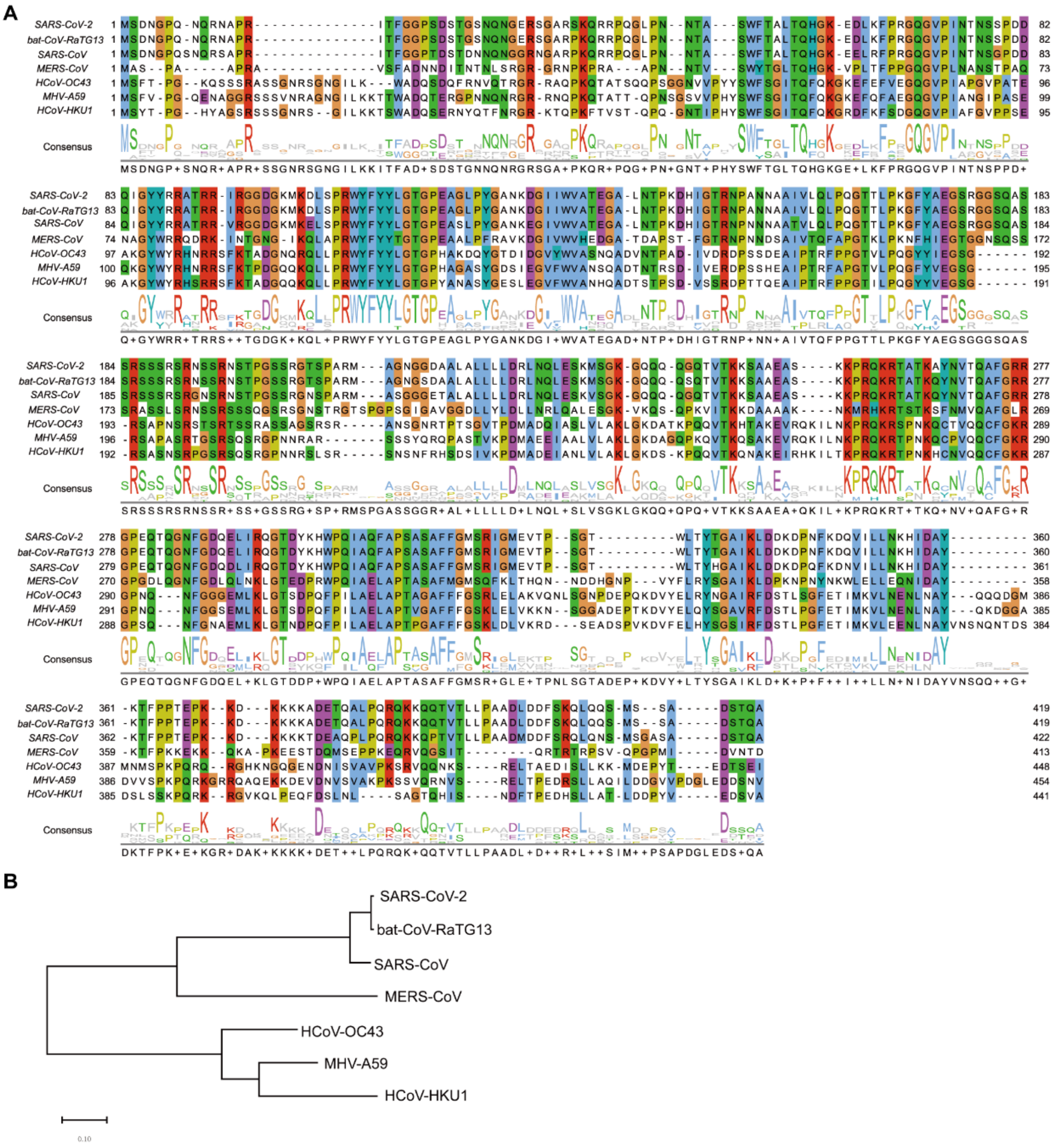

Supplementary Figure S1. Sequence alignment and phylogenetic characterization of N proteins from different coronaviruses. (**A**) Amino acid sequence alignment of N proteins from different coronaviruses. GenBank Accession Numbers were: SARS-CoV, AY394850.2; SARS-CoV-2, YP_009724397.2; bat-CoV-RaTG13, QHR63308.1; HCoV-OC43, YP_009555245; HCoV-HKU1, YP_173242; MERS-CoV, YP_009047211.1; MHV-A59, ATN37902.1. Amino acids are color coded according to similar physicochemical properties. (**B**) Phylogenetic tree of N proteins from related coronaviruses. The tree is drawn using the Neighbor-Joining method with default parameters and bootstrap value 1000 in Mega 7.0.

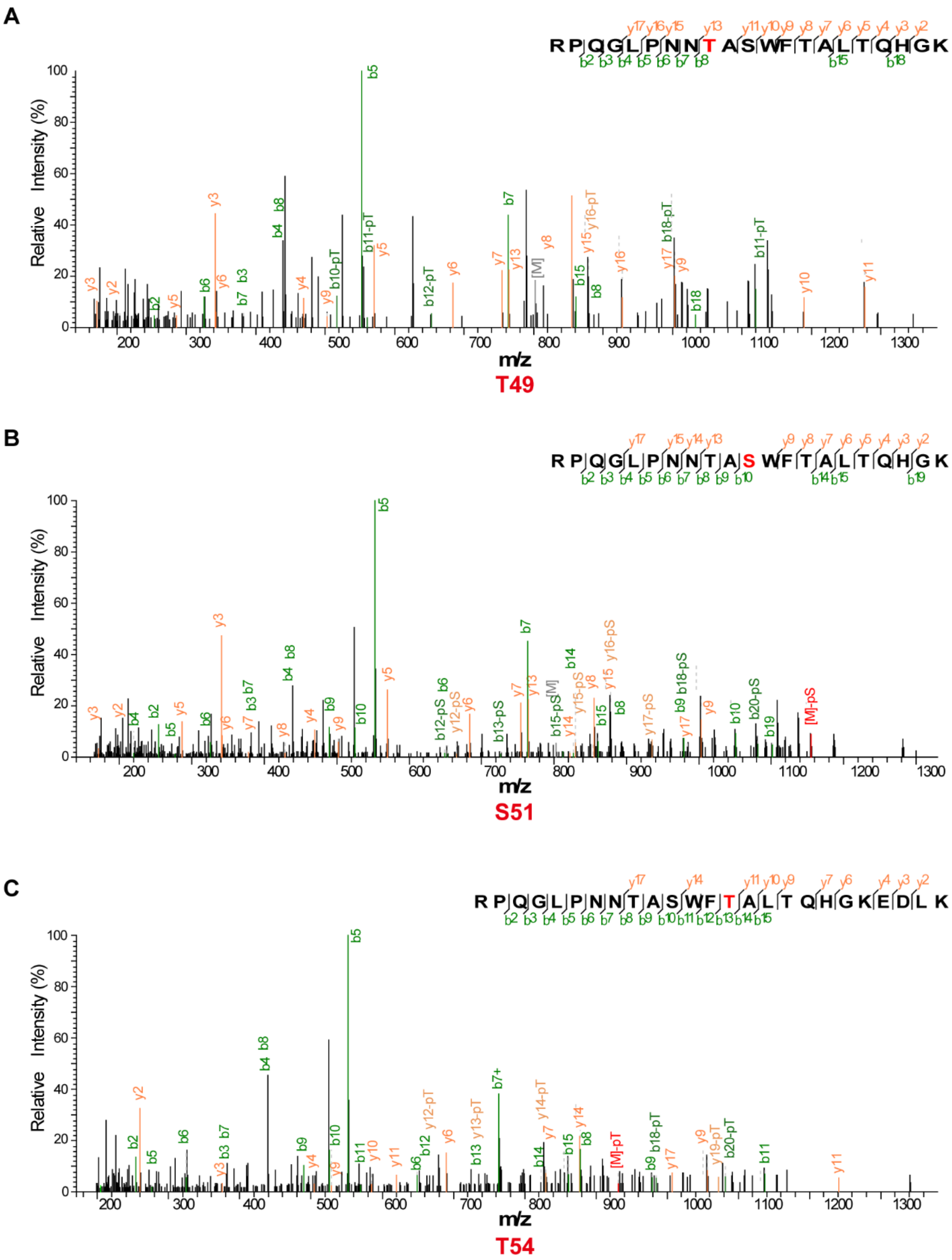
A
RPQGLPNNTASWFTALTQHGK
Relative Intensity (%)
m/z
T49
B
RPQGLPNNTASWFTALTQHGK
Relative Intensity (%)
m/z
S51
C
RPQGLPNNTASWFTALTQHGKEDLK
Relative Intensity (%)
m/z
T54

D

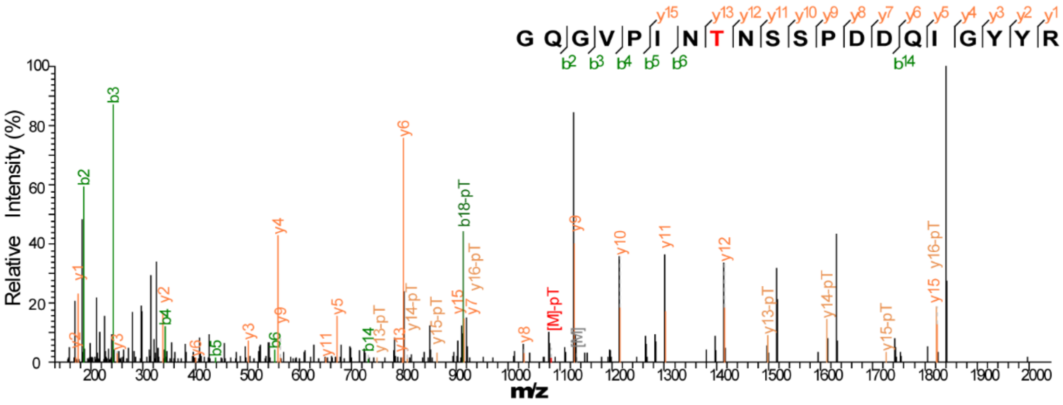

T76phos

E

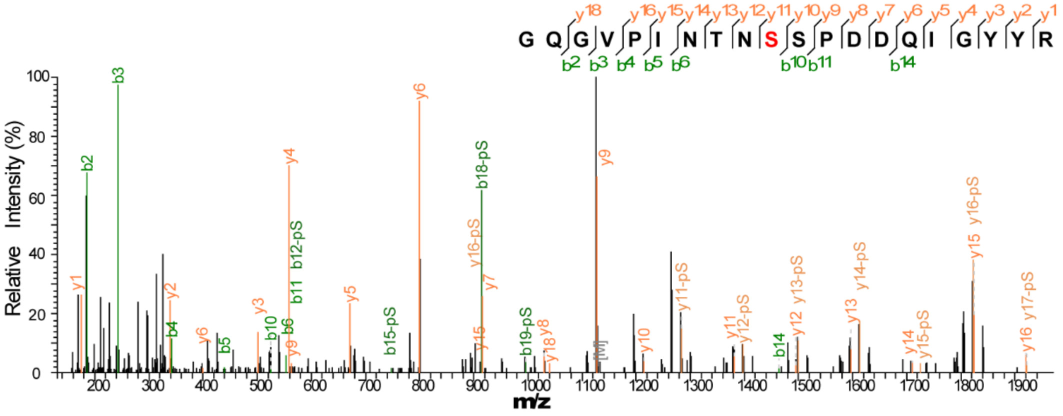

S78phos

F

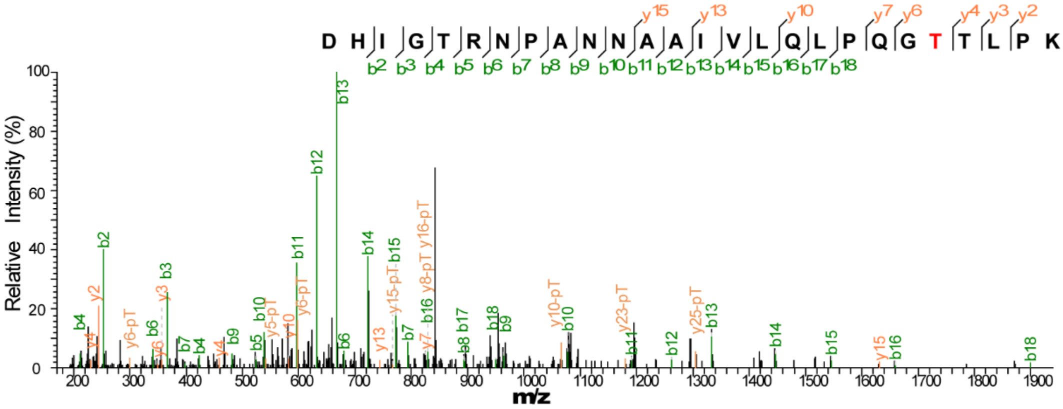

T165phos

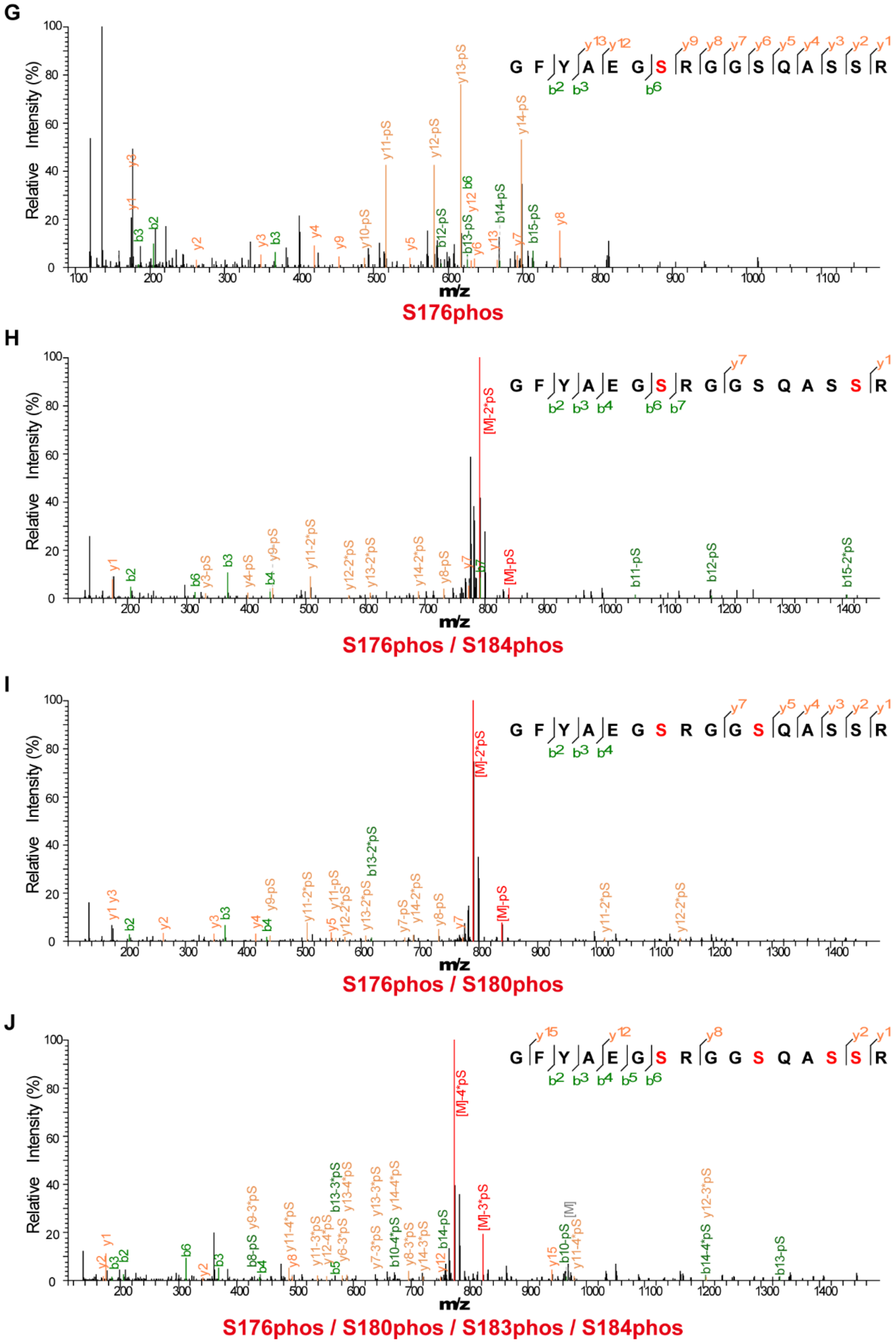
G
Relative Intensity (%)
m/z
G F Y A E G S R G G S Q A S S R
S176phos
H
Relative Intensity (%)
m/z
G F Y A E G S R G G S Q A S S R
S176phos / S184phos
I
Relative Intensity (%)
m/z
G F Y A E G S R G G S Q A S S R
S176phos / S180phos
J
Relative Intensity (%)
m/z
G F Y A E G S R G G S Q A S S R
S176phos / S180phos / S183phos / S184phos

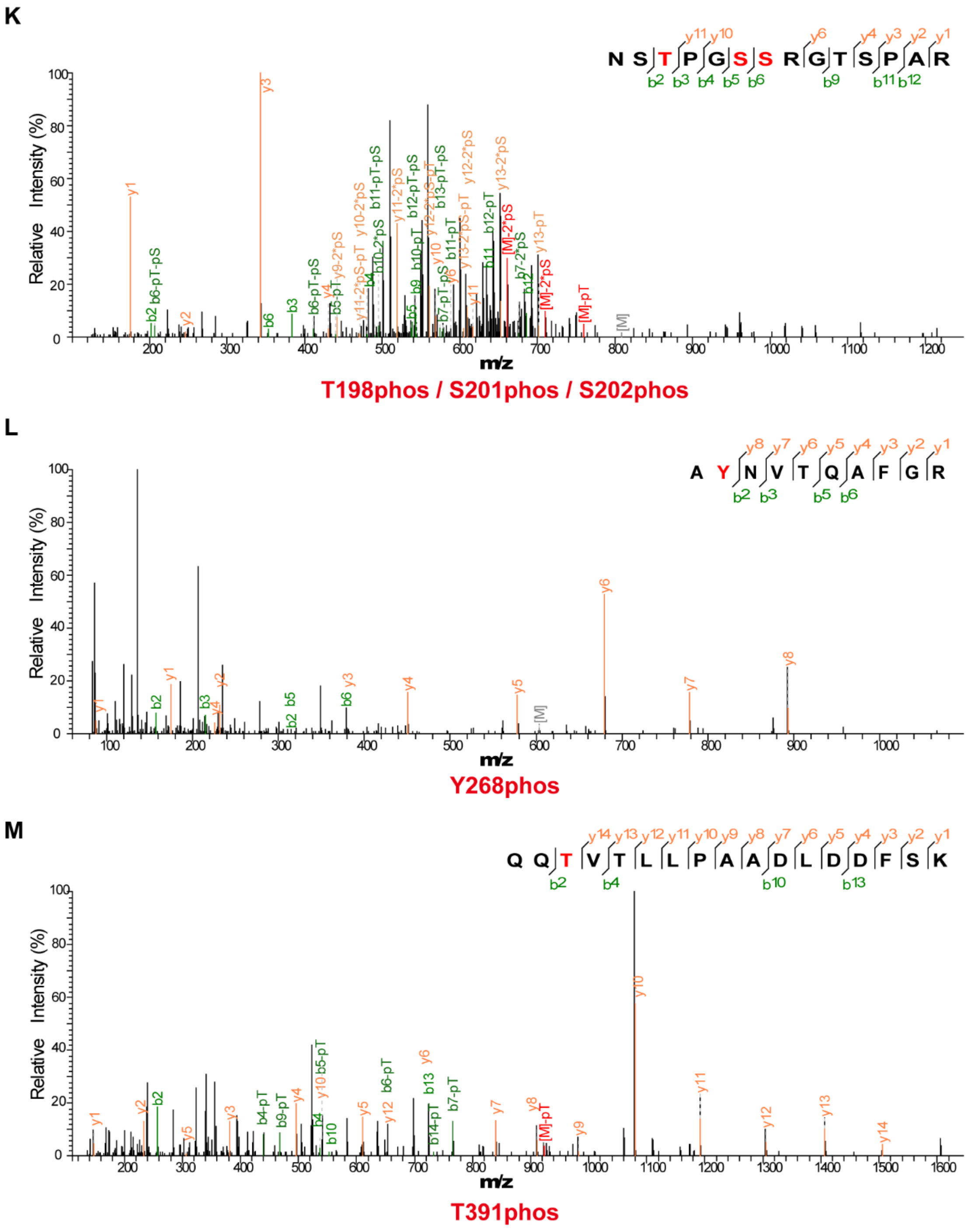

Supplementary Figure S2. Representative MS spectrums of identified phosphorylation sites containing peptides from N protein.

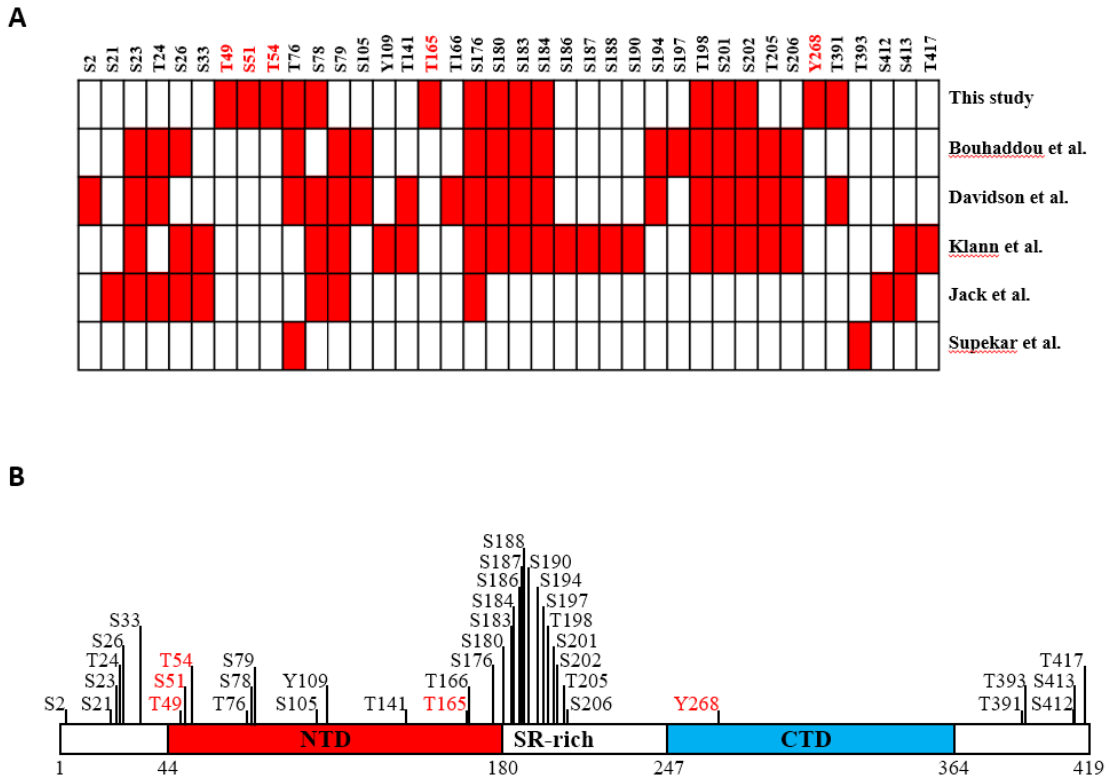

Supplementary Figure S3. Integration analysis of the phosphorylation sites of N protein reported in the literature and identified in the present study. (**A**) The details of the reported phosphorylation sites(Bouhaddou et al., 2020; Davidson et al., 2020; Jack et al., 2020; Klann et al., 2020; Supekar et al., 2021). (**B**) The positions of the phosphorylation sites were represented in the schematic diagram of N protein. The previously unreported phosphorylation sites are marked in red.

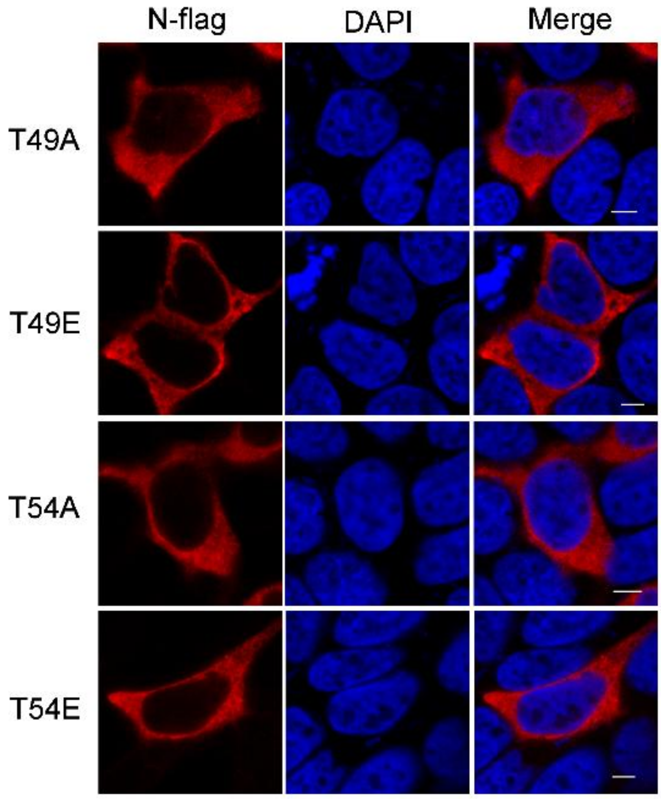

Supplementary Figure S4. Subcellular localization of N protein mutations in T49 and T54. The Caco-2 cells were transfected with the indicated plasmids and then were fixed and stained with anti-flag antibody (red) and DAPI (blue) 24 hours later. Scale bar, 10 μm.

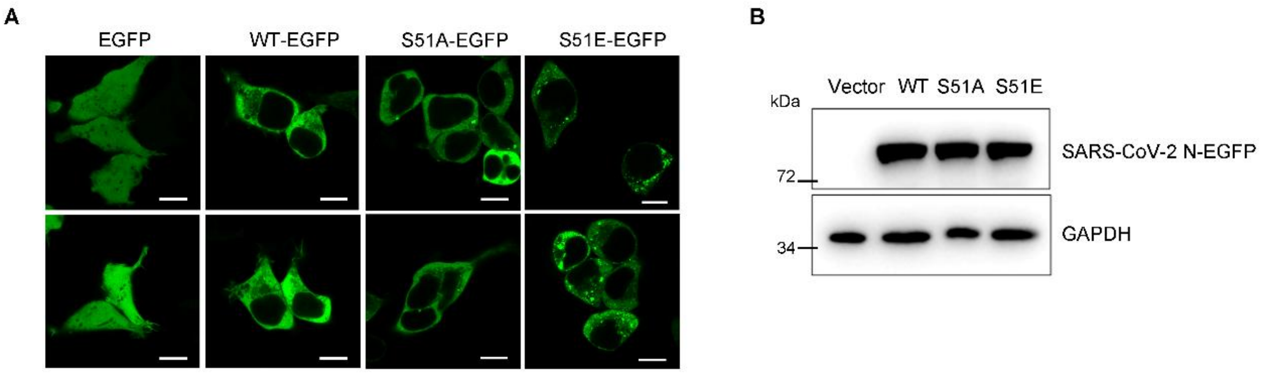

Supplementary Figure S5. The S51E mutant of N protein forms puncta in 293T cells. (**A**). The 293T cells were transfected with the indicated plasmids and then images were taken under a Zeiss LSM 880 Confocal Microscope 24 hours later. Scale bar, 10 μm. (**B**). The expression level of N mutants in 293T cells were measured by Western Blot with the indicated antibodies.

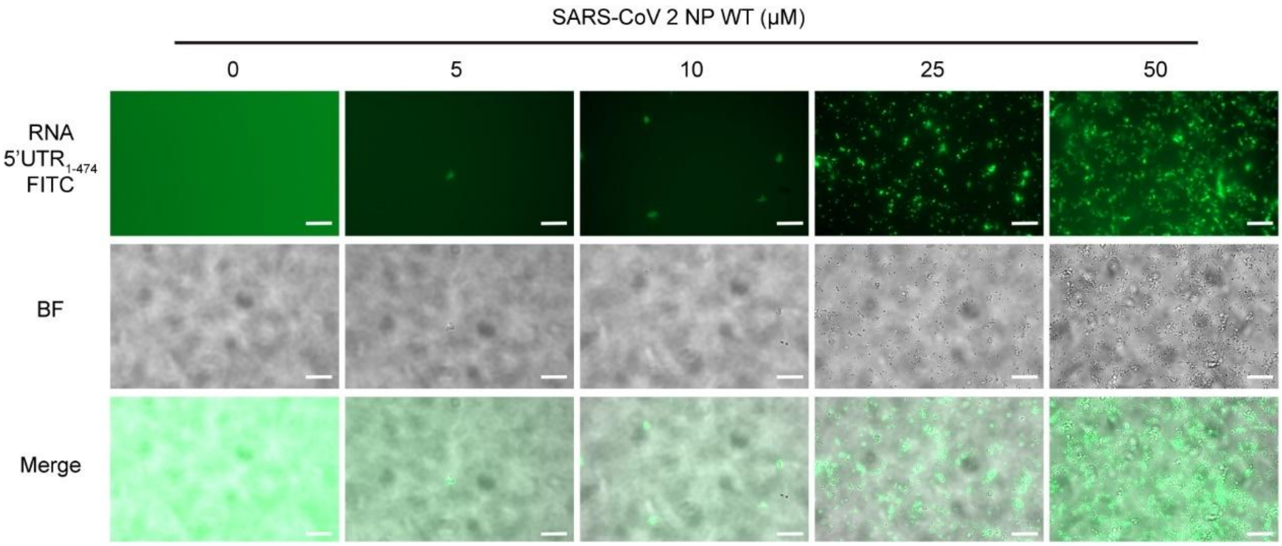

Supplementary Figure S6. N protein of SARS-CoV-2 undergoes phase separation with viral RNA. The indicated concentrations of N protein were mixed with FITC-labeled 5' UTR of viral RNA at 37 °C. Fluorescence and bright-field (BF) images were taken after 20 min of incubation. Scale bar, 10 μm.

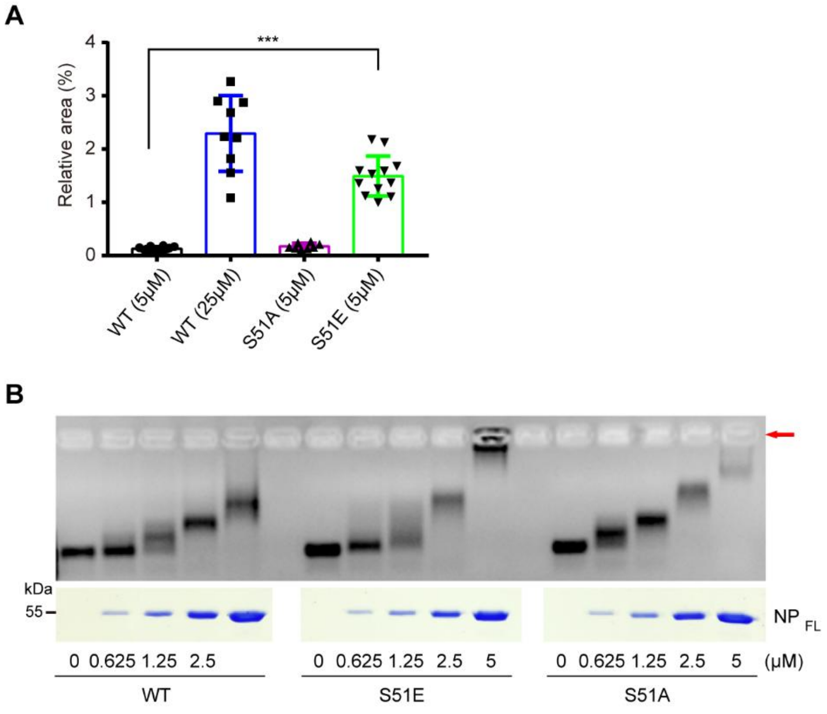

Supplementary Figure S7. The S51E mutation promotes the phase separation of N protein *in vitro*. (**A**) Comparison of the percentage of area occupied by droplets in each field of view in Fig. 1**N**. The area of droplet occupied in the image was measured with ImageJ software. The representative data are shown as the mean ± SD (n=10, 9, 7, 12). Statistical analyses were performed using Mann-whitney U test. (**B**) The indicated concentrations of N protein (WT, S51A or S51E mutants) were mixed with 5' UTR (1-400 nt) of viral RNA and incubated for 20 min at 37 ℃. The protein and RNA mix were loaded and separated in 1.5% agarose gel and stained with EtBr.

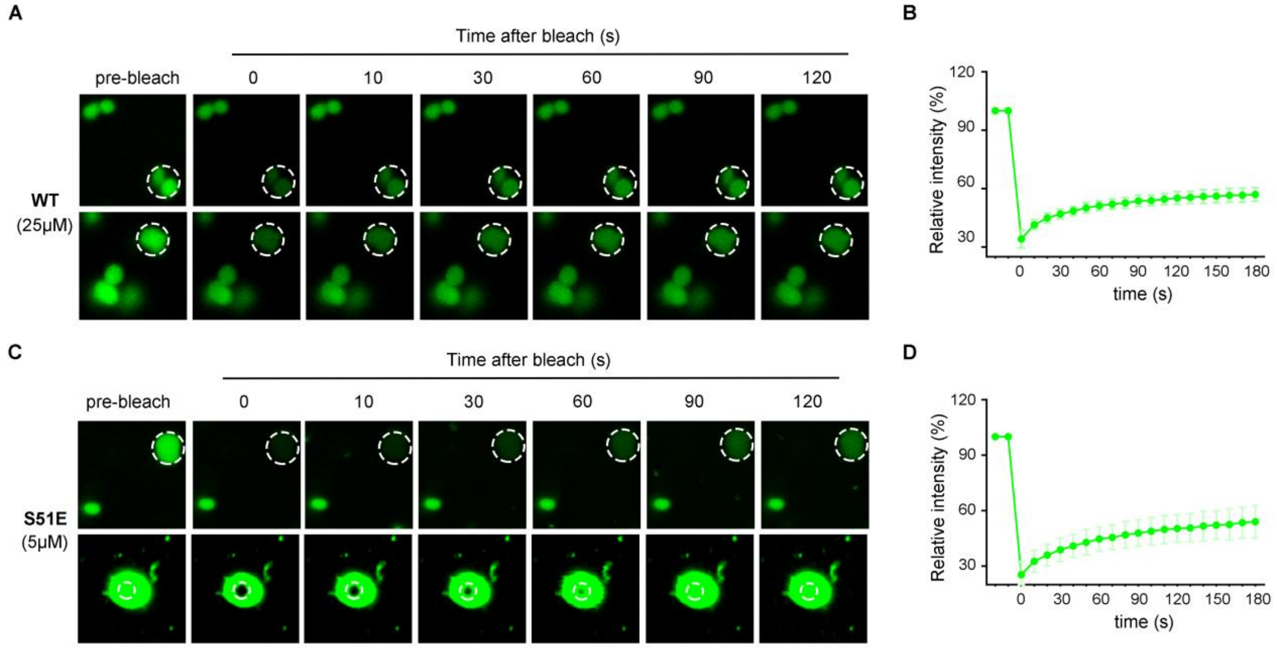

Supplementary Figure S8. FRAP analysis of droplets formed by N protein *in vitro*. The WT (25 μM) or S51E mutant (5 μM) N protein were incubated with FITC-labeled 5' UTR of viral RNA and incubated for 20 min at 37 ℃. The droplets were photobleached and time-lapse imaging were performed (**A** and **C**). The photobleached areas are marked by white circles. The relative fluorescence intensity to pre-bleaching intensity was calculated (**B** and **D**) (mean ± SD, n = 9).

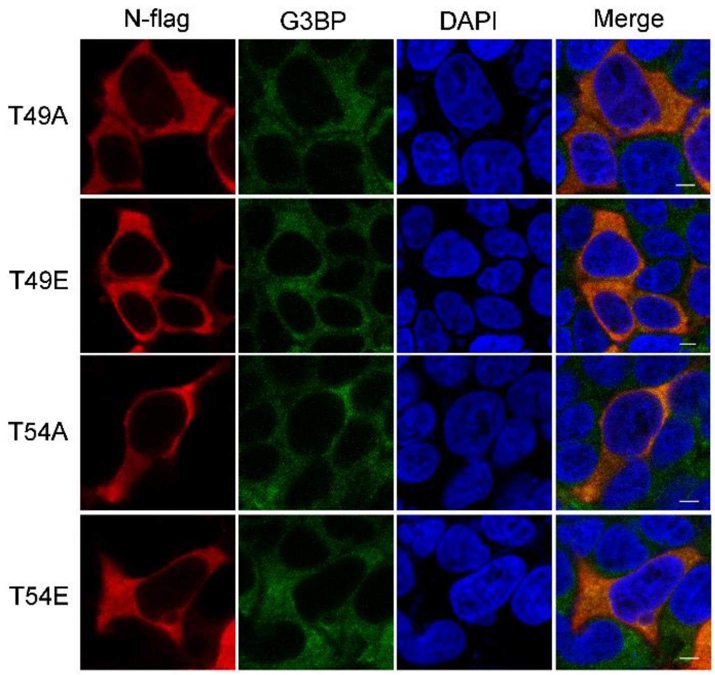

Supplementary Figure S9. Mutations in T49 and T54 of N protein were colocalized with G3BP, but did not induce the formation of stress granule. The Caco-2 cells were transfected with the indicated plasmids for 24 hours later. Then cells were fixed with 4% PFA and co-immunostained with anti-flag (red) and anti-G3BP (green) antibodies. Nuclei were stained by DAPI (blue). Scale bar, 10 μm.

Supplementary Tables

Supplementary Table S1. The identified phosphorylated peptides in N protein in this study.

Position	Phosphorylated peptide	Number
**T49**	RPQGLPNN**T**ASWFTALTQHGK	11
**S51**	RPQGLPNNTA**S**WFTALTQHGK	11
**T54**	RPQGLPNNTASWF**T**ALTQHGKEDLK	11
**T76**	GQGVPIN**T**NSSPDDQIGYYR	10
**S78**	GQGVPINTN**S**SPDDQIGYYR	20
**T165**	DHIGTRNPANNAAIVLQLPQG**T**TLPK	9
**S176**	GFYAEG**S**RGGSQASSR	20
**S176/ S184**	GFYAEG**S**RGGSQAS**S**R	10
**S176/ S180**	GFYAEG**S**RGG**S**QASSR	10
**S176/ S180/ S183/ S184**	GFYAEG**S**RGG**S**QA**SS**R	20
**T198/ S201/ S201**	NS**T**PG**SS**RGTSPAR	9
**Y268**	A**Y**NVTQAFGR	12
**T391**	QQ**T**VTLLPAADLDDFSK	9

Supplementary Table S2. The conservation of the phosphorylation sites in N protein.

Position	Number of mutations	% of mutation (n=57613)	% of conservation (n=57613)
S2	1	0.002	99.998
S21	2	0.003	99.997
S23	7	0.012	99.988
T24	58	0.101	99.899
S26	6	0.010	99.990
S33	59	0.102	99.898
T49	0	0.000	100.000
S51	0	0.000	100.000
T54	0	0.000	100.000
T76	8	0.014	99.986
S78	0	0.000	100.000
S79	3	0.005	99.995
S105	0	0.000	100.000
Y109	0	0.000	100.000
T141	10	0.017	99.983
T165	0	0.000	100.000
T166	16	0.028	99.972
S176	1	0.002	99.998
S180	110	0.191	99.809
S183	180	0.312	99.688
S184	3	0.005	99.995
S186	11	0.019	99.981
S187	39	0.068	99.932
S188	517	0.897	99.103
S190	157	0.273	99.727
S194	1776	3.083	96.917
S197	654	1.135	98.865
T198	10	0.017	99.983
S201	10	0.017	99.983
S202	344	0.597	99.403
T205	162	0.281	99.719
S206	55	0.095	99.905
Y268	0	0.000	100.000
T391	21	0.036	99.964
T393	0	0.000	100.000
S412	6	0.010	99.990
S413	11	0.019	99.981
T417	9	0.016	99.984
S441	0	0.000	100.000

Supplementary Table S3. The mutation details of the phosphorylation sites in N protein.

protein_id	name	aa_change	summary
YP_009724397.2	N	S2Y	1
YP_009724397.2	N	S21L	2
YP_009724397.2	N	S23L	4
YP_009724397.2	N	S23T	2
YP_009724397.2	N	S23P	1
YP_009724397.2	N	T24N	40
YP_009724397.2	N	T24I	17
YP_009724397.2	N	T24A	1
YP_009724397.2	N	S26C	2
YP_009724397.2	N	S26I	2
YP_009724397.2	N	S26G	1
YP_009724397.2	N	S26N	1
YP_009724397.2	N	S33I	55
YP_009724397.2	N	S33G	3
YP_009724397.2	N	S33N	1
YP_009724397.2	N	T76I	8
YP_009724397.2	N	S79N	3
YP_009724397.2	N	T141I	10
YP_009724397.2	N	T166I	16
YP_009724397.2	N	S176G	1
YP_009724397.2	N	S180I	96
YP_009724397.2	N	S180R	3
YP_009724397.2	N	S180C	3
YP_009724397.2	N	S180T	3
YP_009724397.2	N	S180N	3
YP_009724397.2	N	S180G	2
YP_009724397.2	N	S183Y	179
YP_009724397.2	N	S183T	1
YP_009724397.2	N	S184P	2
YP_009724397.2	N	S184F	1
YP_009724397.2	N	S186F	4
YP_009724397.2	N	S186Y	4
YP_009724397.2	N	S186P	3
YP_009724397.2	N	S187L	39
YP_009724397.2	N	S188L	495
YP_009724397.2	N	S188P	22
YP_009724397.2	N	S190I	127
YP_009724397.2	N	S190G	29
YP_009724397.2	N	S190N	1
YP_009724397.2	N	S194L	1768
YP_009724397.2	N	S194A	8

YP_009724397.2	N	S197L	652
YP_009724397.2	N	S197T	1
YP_009724397.2	N	S197F	1
YP_009724397.2	N	T198I	10
YP_009724397.2	N	S201G	5
YP_009724397.2	N	S201C	4
YP_009724397.2	N	S201N	1
YP_009724397.2	N	S202N	333
YP_009724397.2	N	S202C	4
YP_009724397.2	N	S202I	4
YP_009724397.2	N	S202G	3
YP_009724397.2	N	T205I	161
YP_009724397.2	N	T205S	1
YP_009724397.2	N	S206F	52
YP_009724397.2	N	S206P	2
YP_009724397.2	N	S206C	1
YP_009724397.2	N	T391I	21
YP_009724397.2	N	S412C	1
YP_009724397.2	N	S412G	1
YP_009724397.2	N	S412R	1
YP_009724397.2	N	S412I	1
YP_009724397.2	N	S412N	1
YP_009724397.2	N	S412T	1
YP_009724397.2	N	S413I	9
YP_009724397.2	N	S413N	2
YP_009724397.2	N	T417I	8
YP_009724397.2	N	T417A	1

Supplementary Table S4. List of primers used in this study.

Target name	Forward primer	Reverse primer
**For point mutation**		
T49A	GGTTTACCCAATAATGCTGCGTCTTGGTTCA	TGAACCAAGACGCAGCATTATTGGGTAAACC
T49E	GGTTTACCCAATAATGAGGCGTCTTGGTTCACC	GGTGAACCAAGACGCCTCATTATTGGGTAAACC
S51A	CCCAATAATACTGCGGCTTGGTTCACCGCTC	GAGCGGTGAACCAAGCCGCAGTATTATTGGG
S51E	CCCAATAATACTGCGGAGTGGTTCACCGCTCTC	GAGAGCGGTGAACCACTCCGCAGTATTATTGGG
T54A	ACTGCGTCTTGGTTCGCCGCTCTCACTCAAC	GTTGAGTGAGAGCGGCGAACCAAGACGCAGT
T54E	ACTGCGTCTTGGTTCGAGGCTCTCACTCAACAT	ATGTTGAGTGAGAGCCTCGAACCAAGACGCAGT
**pHAGE-CMV-Flag subclone**		
Full-length	atttGCGGCCGCgccaccATGTCTGATAATGGACCCCA	CGGCTAGCTTACTTGTCATCGTCGTCTTTGTAGTCGGCCTGAGTTGAGTCAG
**pET-30a (+) subclone**		
Full-length	cgcGGATCCTCTGATAATGGACCCCA	atttGCGGCCGCTTAGGCCTGAGTTGAGTCAGCACTGCT
NTD 1-180	cgcGGATCCTCTGATAATGGACCCCA	atttGCGGCCGCTTAACTGCCGCCTCTGCTCCCTTCTGC
**For in vitro transcription**		
5’ UTR 1-474	TAATACGACTCACTATAGGGATTAAAGGTTTATACCTTCCCAG	AACACATAGGGCTGTTCAAGTTG
**For qPCR**		
5’UTR	CCTTGTCCCTGGTTTCAACG	AGATGTTGACGTGCCTCTGA
